# Synergistic rhizosphere degradation of γ-hexachlorocyclohexane (lindane) through the combinatorial plant-fungal action

**DOI:** 10.1371/journal.pone.0183373

**Published:** 2017-08-31

**Authors:** Michael Dare Asemoloye, Rafiq Ahmad, Segun Gbolagade Jonathan

**Affiliations:** 1 Food and Environmental Mycology/Biotechnology Unit, Department of Botany, University of Ibadan, Ibadan, Nigeria; 2 Department of Environmental Sciences, COMSATS Institute of Information Technology, Abbottabad, Pakistan; MJP Rohilkhand University, INDIA

## Abstract

Fungi are usually involved in degradation/deterioration of many anthropogenic wastes due to their verse enzyme secretions and adaptive capabilities. In this study, five dominant fungal strains were isolated from an aged lindane polluted site, they were all mixed (100 mg each) together with pent mushroom compost (SMC) and applied to lindane polluted soil (5 kg) at 10, 20, 30, 40% and control 0% (soil with no treatment), these were used to grow *M*. *maximus* Jacq for 3 months. To establish lindane degradation, deductions such as Degradation rate (K_1_), Half-life (t_1/2_) and Degradation efficiency (DE) were made based on the analyzed lindane concentrations before and after the experiment. We also tested the presence and expressions of phosphoesterases (*mpd* and *opd-A*) and catechol 1,2-dioxygenases (*efk2* and *efk4*) genes in the strains. The stains were identified as *Aspergillus niger* (KY693970); *Talaromyces atroroseus* (KY488464), *Talaromyces purpurogenus* (KY488468), *Yarrowia lipolytica* (KY488469) and *Aspergillus flavus* (KY693973) through morphological and molecular methods. Combined rhizospheric action of *M*. *maximus* and fungi speed up lindane degradation rate, initially detected lindane concentration of 45 mg/kg was reduced to 11.26, 9.34 and 11.23 mg/kg in 20, 30 and 40% treatments respectively making 79.76, 85.93 and 88.67% degradation efficiencies. K_1_ of 1.29 was recorded in control while higher K_1_ of 1.60, 1.96 and 2.18 /day were recorded in 20, 30 and 40% treatments respectively. The best t_1/2_ of 0.32 and 0.35 /day were recorded in 40 and 30% compared to control (0.54 /day). All the strains were also affirmed to possess the tested genes; *opd* was overexpressed in all the strains except KY693973 while *mpd* was overexpressed in KY693970, KY488464 but moderately expressed in KY488468, KY488469 and KY693973. However, *efk* genes were under-expressed in most of the strains except KY488469 and KY693973 which showed moderate expression of *efk4*. This work suggests that the synergistic association of the identified rhizospheric fungi and *M*. *maximus* roots could be used to remove lindane in soil at a limited time period and this combination could be used at large scale.

## Introduction

Pesticides use in agriculture is important as they are effective in pest and disease control management, but excessive use of many pesticides as affected food security and concurrent health threats in humans [1, 2]. Most of these pesticides are recalcitrant organic compounds which are not easily degraded by natural means and they are referred to as persistence organic pollutants (POPs). POPs are of two types, the organophosphate pesticides and the organochlorine pesticides, they are of great environmental and health concerns due to their toxic, persistence and bio-accumulative capacities [3], many of them may form residual compounds which are more toxic in the soil and can get accumulated in living tissue through direct or indirect means, by this they can get into the food chain of an ecosystem and affect wide range of organisms.

A good example of an organochlorine pesticide is lindane. Lindane is a common name for 1r, 2R, 4r, 5r, 3S, 6s-Hexachlorocyclohexane; g-isomer, it is also known under several trade names such as aalindane, aficide, agrisol G-20, agrocide, agrone xit, gammaline 20 and so on. According to Li et al. [4] lindane is a synthesized pesticide which mainly consist about 10–20% gamma γ, 60–79% alpha α, 5–12% Beta β, and 6–10% δ-Isomers and it is called γ-HCH because it is only the γ-isomer has insecticidal activities while the remaining are discarded during purification. It is a very common and in fact one of the most commonly used pesticide for crops insect and disease control [5], in veterinary [6–9]. Its toxicity can travel to long distance on air [10, 11]. It has been classified as carcinogens and injurious to human health [12]. Lindane use was banned or restricted in many countries but due to its effectiveness in killing pests many harmers still use it and it is still used in many developing countries under different trade name and its toxicity still persist in many soils and sites were they were produced or applied and this is still an issues of concerns as these sites need to be remediated [13].

Lindane usually degrades under aerobic and anaerobic environments, but it can be mineralized only in aerobic condition [14, 15]. Many bacteria has been reported for lindane degradation [15–18] and many scientist had attributed this to their tolerance and development of enzyme and genetic mechanisms to mineralize the pesticide over the years [15]. Enzymes and regulatory genes involved in lindane degradation are of immense importance in soil clean-up from lindane pollution, these has been well studied in *Sphingomonas* species example is *Sphingobium japonicum* UT26 which was isolated from lindane polluted soil for almost 12 years [19]. In another study, it was reported that this UT26 was able to mineralize lindane as a sole carbon and energy source [20]. Lindane degradation and mineralization was also reported by other species of *Sphigobium* such as the Indian strains of *S*. *indicum* B90 [21], B90A [22] and the France strain *S*. *francense* Sp+ [23]. Pathways was proposed for lindane degradation by *Sphingobium japonicum* UT26 [19] and it was associated to be driven by Lin genes in the organism which was since then believed to be similar in all the other strains of *Sphingobium* with lindane degrading capability. However, Phillips et al. [15] reported an extensive information on general microbial degradation for this pesticide and associated it with diverse and different genes, some other details were reported by Lal et al. [24] and Nagata et al. [25] in *Sphingomonads*. However, little is known about fungal degradation of lindane and there are more possibilities that fungi would be able to adequately degrade lindane based on their wide range of enzyme secretions.

In this article, synergistic capabilities of different lindane utilizing fungi strains isolated from lindane degraded soil was studied, their tolerance capacities were accessed and as well lindane degrading kinetics in terms of degradation efficiency, degradation constant and the half-life were also studied in microcosm experiment and possible lindane degrading partways through which this might be happening were suggested. In addition, different suspected degrading genes were detected in the fungal strains and their expressions were cstudied. Results of this work provide more useful information on the synergistic fungal-root rhizospheric degradation of lindane for better soil clean-up in lindane degraded soils and this as well could be used for the degradation of other organic compounds especially the organochlorine pesticides.

## Materials and methods

### Chemicals and reagents

Reagents used were obtained from Sigma-Aldrich, dichloromethane (DCN), hexa-decyl trimethyl ammonium bromide (cTAB), isopropanol, lithium chloride, tris HCl, TAE (50x), TE, EDTA, NaCl_2_, and lindane (used for tolerance test) were all of analytical grades (at least 99% pure). Master-mix containing *Taq* polymerase, 10 mM Tris z HCl (pH 8.3), and 50 mM KCl, DNA loading dye (5X), gene primers, and Complementary DNA (cDNA) synthesis kit were all enzynomic products.

### Collection of lindane polluted soil samples

An aged lindane polluted soil was identified around sewage discharged tank from an agro-pesticide manufacturing company located in Nigeria (7° 11' 0" N, 5° 35' 0" E), about 5kg of the polluted soil samples were collected from 8 spots at about 2–15 cm depth into soil surfaces. The collected soil samples were pooled to make 40 kg and mix together with clean shovel to properly homogenized, repacked in a black polyethylene bag, soil pH was tested on the homogenized sample on sight and brought into laboratory for further experiment.

### Isolation of lindane degrading fungi strains

Lindane utilizing rhizosphere fungal strains were isolated from the aged polluted site by collecting polluted soil (10 g each) from 50 different spot around the root of grasses growing on the soil and kept in a sterile container and brought into the laboratory for fungal isolation. Each of the collected 10 g soil sample was subjected to serial dilution (10^−3^) and this was streaked into a sterile prepared potato dextrose agar (PDA) plate and labelled according to the soil sample from which it was inoculated, and then incubated at 25°C for 4–7 days. Mix fungal cultures were separated by sub-culturing them into a new PDA plate to have a single pure fungus strain.

Each isolated fungal strain was subjected to percentage incidence based on its number of times it occurs in the soil samples and this was calculated according to the equation below [26]:

$$\text{Percentage incidence}=\;\frac{Number\;of\;of\;fungal\;spcieccie\;isolated}{Total\;Number\;of\;fungal\;species\;isolatea}\;X\;100$$ (1)

Fungi with more than 50% percentage incidence were considered to be the most surviving ones (dominant) as shown in supporting document S1 Table and were only considered and further studied, these fungal strains were characterized based on morphological, microscopic and molecular characteristics.

### Collection of spent mushroom substrate (SMC)

Spent mushroom compost (SMC) was used as bioaugmentation agent to enhance the fungal survival in the soil during the experiment. SMC was produced from the cultivation of *Pleurotus ostreatus* (an oyster mushroom) at the Mycology laboratory of the University of Ibadan, Nigeria (7.4463°4N, 3.9033°8E). The harvested SMC was air dried and used for the rhizosphere lindane degradation experiment.

### Test plant *Megathyrsus maximus* Jacq.

*M*. *maximus* commonly known as guinea grass was used as a test plant to affirm the rhizosphere lindane degradation during the experiment. This plant was chosen due to its extensive root system and as it is a common grass, the seedlings (germination index of 98%) were first raised in a pot for two weeks and the young plants of equal length and weight were transplanted into the prepared experimental pots.

### Pre and post analysis of lindane polluted soil

The collected polluted soil was analyzed for its pH, Cation exchange capacity (CEC), organic carbon and some other nutrient contents before and after the experiment. Lindane quantity in the soil was as well analyzed. 10 g was removed from pooled and homogenized soil sample and used for pre-chemical analysis, the pH was determined on site with the use of Eutech EcoTestr (pH meter). Macro and micro-nutrient analysis were done according to the methods of Beazley et al [27], the soil total nitrogen (N) was determined using Kjeldahl method by grounding sieved soil sample in aqua regia with nitric acid and hydrochloric acid in 1:3, total phosphorus was determined by molibdophosphoric yellow color method, while the total K, Ca, Mg, and Fe contents were determined by the use of Perkin Elmer Atomic Absorption Spectrophotometer (AAS) 800B (Wellesley, MA), with operational conditions in accordance specifications for the machine usage.

Initial and final lindane concentration in the soil was detected before and after the rhizospheric lindane degradation experiment, this was done according to the procedures of Beazley et al. [27] using GC/MS (PerkinElmer Clarus 8085). The machine is programmable with split or split-less injector with 2mm i.d deactivated fused-silica liner injector port and helium carrier gas that have programed velocity of 30 cm/sec. The soil sample was digested with none polar dichloromethane (DCN) and the extract was collected as supernatant after 6,000 rpm centrifugation for 10 min at 4°C. 1 μl of sample were injected into the machine after preset with ovum conditions set at 275°C of injector-port temperature (isothermal), 80°C initial oven temperature having no hold, then ramped to 290°C at 20°C/min with 4.5 min hold, Mass spectrometer transfer line and ion source heated to 275°C total oven program was 15 min with less than 20 min of injection-to-injection time.

### Lindane tolerance test

The selected dominant fungal strains were tested for their abilities to survive and tolerate lindane on solid PDA. Each of them was grown in 8 cm diameter petri dish plates containing different concentrations of lindane in nutrient medium and their radial growth measured at different time interval Anaisell et al. [28]. Lindane concentrations of 5, 10, 15 and 25% of the medium (v/v) were mixed with 30 ml of the medium in each of the plates before inoculation. The strains were then point inoculated at the center of the plate with 5 μl solution of 1 X 10^4^ spores (/mil) and incubated at 30°C in three replicates. Radial extension rate was measured at every 24 hrs for 16 days to assert the tolerance of the fungi to lindane concentrations, plates without lindane were considered as control.

### Synergistic rhizosphere degradation of lindane

#### Experimental design and layout

The homogenized soil sample was first sterilized after the pre-analysis procedures to avoid interference from other organisms, and there after potted in 5 kg per pot. Pure cultures of the five selected dominant fungal strains were mixed together with the SMC, this mixture was then used as supplement mixed with the soil sample at different concentrations, they were all mixed thoroughly with the soil to homogenize and the test the plant was transplanted into the pots. The treatment was arranged as follows:

- Soil alone (5 kg) + Plant alone = Phytoremediation (0%_1_) Control 1
- Fungi (100 mg each) + SMC (5 kg) + soil (5 kg), no plant = Mycoremediation (0%_2_) Control 2
- Fungi (10 mg each) + SMC (0.5kg) + soil (5 kg) + plant = Synergistic rhizosphere remediation (T1)
- Fungi (20 mg each) + SMC (1 kg) + soil (5 kg) + plant = Synergistic rhizosphere remediation (T2)
- Fungi (35 mg each) + SMC (1.5kg) + soil (5 kg) + plant = Synergistic rhizosphere remediation (T3)
- Fungi (50 mg each) + SMC (2.5kg) + soil (5 kg) + plant = Synergistic rhizosphere remediation (T4)

The set-up was done in three replicates and the pots were arranged on the field with 25cm spacing, field capacity was maintained by daily watering with 100ml of distilled water for 90 days and the experiment was terminated. The treated soils were then subjected to post-analysis.

### Morphological characterization of the lindane degrading fungi

The selected fungal strains were studied for their morphological characters and compared with other already identified strains, the growth pattern such as the color of spores, mycelial color on the plate surface and underside appearance, colony serration, colony diameter, shape of the vesicle, the shape of conidia head, conidiophore structures were studied and the microscopic figures were taken using Olympus photomicrograph (BX51).

### Molecular characterization of the lindane degrading fungi

#### DNA isolation

The fungal genomic DNA was extracted using the method of Kostadinova et al. [29] and Manasiev et al. [30] with some major modifications in other to simplify and reduce the extraction time, cost and stress. Liquid nitrogen, marcaptoethanol, and chloroform isoamyl-alcohol were not used in this procedure. This DNA extraction was done using only hexa-decyl trimethyl ammonium bromide (CTAB) buffer and Isopropanol, prepared CTAB buffer contained 50mM Tris Buffer pH 8.0, 100mM EDTA and 150mM NaCl_2_. 400mg of a 4 day old fungal mycelium was harvested from the nutrient medium and frozen in -80°C freezer for 6 hrs, the frozen mycelium was thereafter grounded in frozen crucible for about 30 sec and recovered into Eppendorf tube which contain pre-warmed 600 μl CTAB extraction buffer (65°C), the CTAB and fungal mycelium mixture was briefly vortexed for about 1 min and kept in -20°C for about 15–20 min, the mixture was then centrifuged at centrifuged at 6,000 rpm for 10 min at 4°C. The supernatant was decanted directly into another Eppendorf tube containing 400 μl of cold isopropanol for DNA precipitation and kept in -20°C for 6 hrs. The isopropanol mixtures was then again centrifuged at 12,000 rpm for 15 min, 4°C and the supernatant was discarded while the DNA pellet was washed with 70% ethanol and dried completely for about 15 min. The pellet was then dissolved in 30–60 μl TE buffer depending on the quantity of pellet recovered and treated with RNase. Quantity of extracted gDNA was checked by loading 1 μl of dissolved DNA on UV NANODROP Spectrophotometer at absorbance ratio of 260nm and 280nm while DNA quantity was checked for each strain using 1% agarose gel electrophoresis containing ethidium bromide.

#### PCR amplification, sequencing and identification

To identify the isolated fungal strains, the Internally Transcribed Spacer (ITS) amplification of the 18s (1609–1627) and 28s (287–266) genes [31, 32] was used. The primers used for this purpose were:

- pITS4-F (5’-TCCGTAGGTGAACCTGCCG-3’) and
- pITS4-R (5’-TCCTCCGCTTATTGATATGC-3’)

This primer combination was perfect for the identification of *Ascomycete* and *Deuteromycete* fungi. The working solution of the primers was prepared (0.5 micromolar) and they were used for the gene amplification in 20 μl reaction volume containing master mix (10 μl), both forward and reverse primers (1 μl each), fungal gDNA template (2 μl) and deionized water (6 μl). The mixture was transferred into thermal cycler for PCR-amplification set at 94°C for 1 min initial denaturation temperature, followed by 35 cycles of 94°C / 45 sec, annealling at 55°C / 1 min followed by 72°C / 1 min and final extension step of 72°C / 8 min and then held at 4°C till infinity. After amplifications, 8 μl of each amplified sample and 2 μl of DNA loading dye (5X enzynomic) were used for gel electrophoresis with 1% agarose and ethidium bromide to check the band intensity of the amplified gene product. The PCR-products were sent (Macrogen, Korea) for sequences, and about 500–800 bp products of the strains were received, registered at NCBI and compared with those available strains in the National Center for Biotechnology Information (NCBI) database using the Basic Local Alignment Search Tool (BLAST) search program. The most similar sequences were compared and phylogenetic dendogram were constructed using MABL (Phylogeny.fr) tool.

### Screening of the fungal strain possessing the putative lindane degrading genes

The isolated fungal strains are believed to possess some abilities in degrading or mineralizing lindane due to their survival and tolerance to lindane pollution over a long period of time. Phosphoesterase genes (*mpd* and *opd-A*) and catechol 1,2-dioxygenase genes (*afk3* and *afk4*) were tested for their presence in the fungal strains, Primers of selected genes shown in Table 1 were used to amplify the genes using the gDNA of each strain. 20 μl reaction mixture was prepared for PCR reaction to check the presence of each selected gene in each strain. The mixture was placed in thermal cycler at PCR conditions prescribed by Paul et al. [33]; 94°C for1 min initial denaturation temperature, followed by 35 cycles of 94°C for 45 sec, annealing temperature are shown in Table 2 for 1 min followed by 72°C for1 min and final extension step of 72°C for 8 min. After amplifications of each gene, 8 μl of each PCR product was mixed with 2 μl of DNA loading dye (5X, enzynomic) for gel electrophoresis in 1% agarose and ethidium bromide to check the quality of the amplified gene product.

**Table 1:** Fungal phosphoesterase and catechol 1, 2-dioxygenase gene-specific sequences for upstream (u) and downstream (d) primers used for RT-PCR amplification.

Enzyme	Primer name	Published Gene	Sequence	Annealing temp (°C)
Phosphoesterase	OPD	*opdA*-u	GATCGTGGATCCCCAATCGGTACAGGCGATCTG	48
		*opdA*-d	GATCGTAAGCTTTTCATCGTTCGGTATCTTGACGGGGAAT	48
Phosphoesterase	MPD	*mpd*-u	AGCAGGTCGACGAGATCTAC	52
		*mpd*-d	TTGACGACCGAGTAGTTCAC	52
Catechol 1,2- dioxygenase	AFK2	*afk2*-u	TCATGCACGGCCGGGTGATC	95
		*Afk2-d*	GGGTGTCGGTCCATGAGCTC	55
Catechol 1,2- dioxygenase	AFK4	*afk4*-u	TCATGCACGGCCGGGTGATC	55
		*Afk*4-d	CTACGCCTGGTCCGCCACCA	55

**Table 2:** Radial extension rate (cm day^-1^) of the fungal strains in plates containing lindane mix with PDA.

Aspergillus strain	Radial extension rate (cm day-1)					% reduction
	0%	5%	10%	15%	20%	
*A*. *niger* asemoC	3.4 ± 0.04a	2.484 ± 0.07b	0.59 ± 0.03c	0.60 ± 0.01c	0.21 ± 0.01c	93.82
*T*. *atroroseus* asemoG	2.21± 0.07a	1.99± 0.06b	1.23± 0.04b	0.65± 0.01c	0.29± 0.005c	86.88
*T*. *purpurogenus* asemoN	0.55 ± 0.022a	0.49 ± 0.04b	0.31 ± 0.01c	0.27 ± 0.01c	0.25 ± 0.01c	54.55
*Y*. *lipolytica* asemoO	4.23±0.67a	3.23±0.59a	1.28±0.23	1.05±0.08b	0.89±0.03b	78.96
*A*. *flavus* asemoP	1.4 ± 0.04a	0.34 ± 0.06b	0.24 ± 0.028c	0.22 ± 0.013c	0.191 ± 0.07d	86.36

Means of the same letter in row are not significantly different from each other (P≥0.5) according to Duncan Multiple range test; means are ± Standard deviation (SD).

### Expressions of putative phosphoesterase and catechol 1,2-dioxygenase genes in selected fungal strains

#### RNA isolation

Total RNA of each fungal strain was first extracted using modified CTAB procedure. Briefly, fungal mycelium (400 mg) was harvested in tubes and frozen for 24 hrs in -80°C, the frozen mycelium was then ground in liquid nitrogen and recovered in Eppendorf tube containing 600 μl CTAB buffer and briefly vortexed for proper mixing, the mixture was kept in -80°C for 6 hrs and then centrifuged at 6, 000 rpm for 8 min, the supernatant was decanted into another Eppendorf containing 3M lithium chloride solution (400 μl) for RNA precipitation [34], this was inverted several times for proper mixing and again kept in -80°C for 6 hrs. The mixture was then centrifuged for 15 min at 12,000 rpm and 4°C and supernatant was discarded. The RNA pellets were washed in 70% ethanol, dried completely and dissolved in 50μl TE buffer. The quantity of RNA extracted in each fungus was checked by loading 1 μl product in UV Spectrophotometer at absorbance ratio of 260nm and 280nm while the RNA quality of each fungus was checked using 1% agarose gel electrophoresis fortified with ethidium bromide. However, in the case of gene expression, RNA quantity was measured on NANODROP and the same RNA quantity were picked from each strain for synthesizing of cDNA to be used for RT-PCR analysis.

#### cDNA synthesis

Complementary DNA (cDNA) to be used for the gene expression was synthesized by preparing 2 ng of each fungal RNA and synthesized into cDNA according the manufacture’s prescription. Each fungal RNA was first added in 1 μl of 100 μM oligo (dT)_18_ and RNAase free water and put in a water bath set at 70°C for 5 min and thereafter kept on ice.. Another mixture was prepared at this time which contains Top script RT buffer (2 μl), dNTP mixture (2 μl), RNAase inhibitor (0.5 μl) and Reverse transcription enzyme (1 μl). This mixture was added into the mixture of oligo (dT)_18_ and RNA mixture after which they were all incubated at 60°C for 60 min, then 95°C for 5 min.

#### Putative lindane degrading gene expression using RT-PCR

1 μl (50 ng) of each fungal cDNA was used for gene expression study using sets for phosphoesterase and catechol 1, 2-dioxygenase genes listed in Table 1. Each gene was amplified in prepared 20 μl reaction volume containing DEPC water (7.4), Master-mix [8.6 μl containing *Taq* polymerase, 10 mM Tris z HCl (pH 8.3), 50 mM KCl], both forward and reverse primers (0.5 mM), and cDNA (50 ng). PCR reaction was performed in thermal cycler at the annealing temperatures (35 cycles) for each primer and PCR conditions described above. The amplified genes PCR product (10 μl) was run on 1.5% agarose gel electrophoresis to determine expressed genes in each strain.

### Statistical data analysis

Quantitative data obtained during this experiment were analyzed with the use of Minitab version 17, analysis of variance (ANOVA) and Duncan Multiple Range Test (*p* ≤ 0.05) were done.

Salient deductions were also made from the quantified data obtained in this study; the equations were deduced from general chemical and physical reaction phenomena as follows:

#### i. Percent degradation (DE)

This was determined by comparing the initial and final concentrations of lindane quantified from the soil before and after the experiment according to the Eq 2 below:

$$\text{DE }\left(\text{\%}\right)=\;\frac{Co-Ct}{Co}\;X\;100$$ (2)

Where Co = Initial lindane concentration of the soil (mg/kg) and Ct = Residual/final total lindane concentration (mg/kg) respectively.

#### ii. Analysis of degradation rate constant (K_1_)

Degradation rate constant (k_1_) was deduced in /day unit according to first order reaction kinetic of chemical reactions:

$$\text{LOG }\left(Co-Ct\right)=\text{Log }Co-\;Log\frac{K1}{2.303}\text{t}$$ (3)

Where Co = Initial lindane concentration (mg/kg), Ct = Final total lindane concentration (mg/kg), at time *t* (/day), K_1_ = lindane degradation rate (day^-1^)

#### iii. Estimation of lindane half-life times

This was deduced from the general half-life formula given in Eq 4 below:

$$t\frac{1}{2}=\;\frac{\text{ln}2}{K1}$$ (4)

Where t_1/2_ is the Half-life, *k* is the biodegradation rate constant (day-1).

## Ethics statement

No specific permits were required for the described sampling and field studies and locations. We confirm that the location is not privately-owned or protected. Also this study do not involve the use of any endangered plant or animal species.

## Results

### Lindane utilizing fungal strains

**Fig 1:**
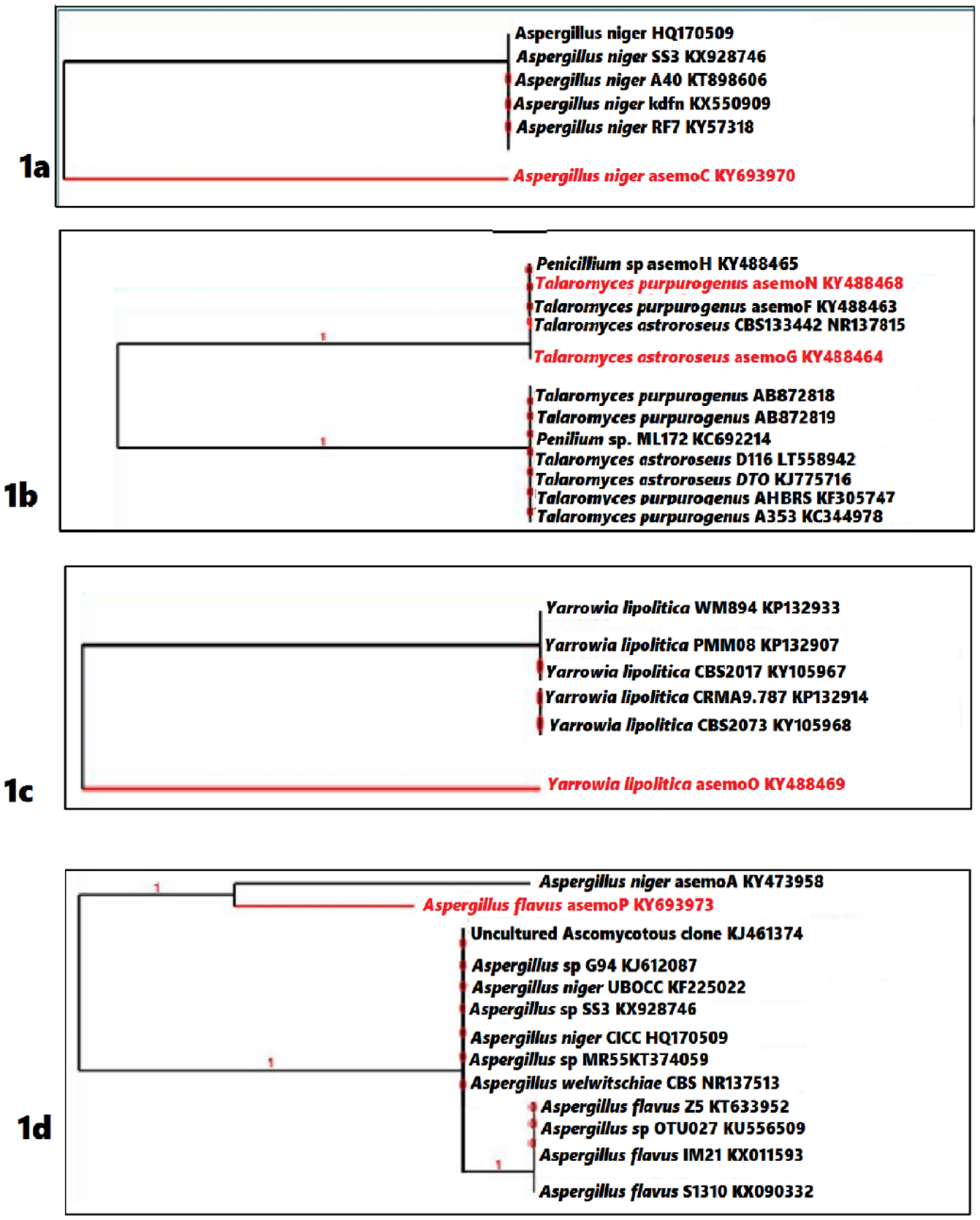
Phylogenetic tree of lindane degrading fungal strains, based on a distance matrix analysis of the ITS sequences. (1a) *Aspergillus niger* asemoC KY693970; (1b) *Talaromyces atroroseus* asemoG KY488464 and *Talaromyces purpurogenus* asemoN KY488468; (1c) *Yarrowia lipolytica* asemoO KY488469; (1d) *Aspergillus flavus* asemoP KY693973.

Fifty (50) total fungal strains were isolated from the lindane polluted sites and five (5) strains had highest incidence of 50% above (S1 Table). These five strains were coded as asemoC (82%), asemoG (56%), asemoN (56%), asemoO (68%), and asemoP (68%). They were hypothesized as the most lindane utilizing fungi as the soil has been exposed to pesticide contamination for over 10 years. These selected strains were thereafter identified as *Aspergillus niger* (asemoC) with accession number KY693970, *Talaromyces atroroseus* (asemoG) KY488464, *Talaromyces purpurogenus* (asemoN) KY488468, *Yarrowia lipolytica* (asemoO) KY488469 and *Aspergillus flavus* (asemoP) KY693973 (S2 Table). The ITS gene amplification (S1 Fig) and the constructed phylogenetic relationship for each strain and its other fungal species showed that, strain asemoC (KY693970) shares common ancestor with previously identified *A*. *niger* HQ170509.1, *A*. *niger* KX928746, *A*. *niger* KT898606.1, *A*. *niger* KX550909.1 and *A*. *niger* KY357318.1 (Fig 1a).

Interestingly, strain *T*. *astroroseus* asemoG KY488464 had high similarity relationships with our previously reported fungal strain *T*. *astroroseus* asemoF KY488463 which was recovered from the crude oil polluted soil, there is high possibilities that this can also act like the lindane utilizing strains KY488464 reported in this work (Fig 1b). Strain asemoN shows high homology with our previously reported *Penicillium* sp. AsemoH KY488465 which was also reported as polyaromatic hydrocarbon degrading fungus (Fig 1b), this strain asemoN also shares common ancestor organism with other *T*. *purpurogenus* strains KF114739, NR137815, AB8728 and *Penicillium* sp. KC692214. Moreover, the strain asemoO KY488469 in Fig 1d showed common ancestral organism with five other strains of *Y*. *lipolytica* KP132933, KP132907, KY105967, KP132914 and strain KY105968. In the same way, strain asemoP KY693973 shared very common similarities with our previously reported *A*. *niger* asemoA which was reported as crude oil degradation fungal strain and they both shares common ancestors with other *Aspergillus* strains as shown in Fig 1e.

It is good to note here that other fungi with high similarities with fungal strains reported in this work may also possess ability to tolerate and or degrade lindane, though most of the similar strains with those reported in this work were reported to degrade crude oil or polyaromatic hydrocarbons, they should be studied for bioremediation of lindane polluted soils with production of similar enzymes.

### Fungal tolerance to lindane

All the five (5) fungal strains used in this study showed high tolerance up to 20% lindane concentrations (Table 2). However, the fungal growth reduced as lindane concentration increased. *Y*. *lipolytical* asemoO tolerated lindane concentration but its growth reduced from 3.23 cm in 5% lindane concentration to 0.89 cm. *A*. *niger* asemoC growth reduced from 2.48 cm in 5% lindane concentration to 0.21 cm in 20% concentration. In the same way *T*. *astroroseus* asemoG growth reduced from 1.99 to 0.29 and *A*. *flavus* reduced from 0.34 cm to 0.19 cm while *T purpurogenus* reduced from 0.49 cm to 0.25 cm. On the basis of percentage reduction, *T*. *purpurogenus* and *Y*. *lipolytica* had the highest tolerance to lindane concentration at 54.55 and 78.96% reduction respectively compared to the other three fungal strains *A*. *flavus*, *T astroroseus* and *A*. *niger* which had 86.36, 86.88 and 93.82% tolerance.

### Synergistic rhizosphere degradation of lindane

The rhizosphere degradation of lindane in this study was showed that the synergistic rhizospheric actions of the five fungal strains, SMC, and *M*. *maximum* roots actively degraded lindane compared to when the soil was treated with each of the component separately. The synergistic system improved the soil nutrients; the pH of the soil was adjusted from 4.9 to 6.9 in 30 and 40% treatments. The lindane concentration of 45.00 mg/kg was reduced in all treatments including the controls after the 3 months experiment. Plant alone (control 1) reduced lindane concentration of 45 mg/kg to 12.34 mg/kg which makes 32.66% loss. Fungal and SMC mixture alone (control 2) reduced the concentration from 45 mg/kg to 11.94 mg/kg which makes 33.06% loss. These results shows that both plant and fungi are capable of lindane degradation as earlier reported by many scientists. In this study however, combinations of all the treatments fungi, SMC and plant degraded more of the lindane concentrations in the soil, it was also observed that the lindane degradation increase and the synergistic treatment increased from T1 to T4. The lindane concentration reduced to 12.11, 9.110, 6.330 and 5.100 mg/kg in T1, T2, T3 and T4, these makes 32.89, 35.89, 38.67 and 39.90% loss of lindane (Table 3). This showed that the T4 (40%) and T1 (30%) had the highest lindane % loss.

**Table 3:** Lindane degradation kinetics during synergistic rhizosphere degradation.

Treatments	Co (mg/kg)	Ct (mg/kg)	LOSS (mg/kg)	DE (% Loss)	K_1_ (day^-1^)	t_1/2_ (/day)
**Control 0%**_**1**_	45.00	12.34	32.66	72.58	1.291	0.536
**Control 0%**_**2**_	45.00	11.94	33.06	73.47	1.371	0.506
**T1**	45.00	12.11	32.89	73.09	1.313	0.528
**T2**	45.00	9.110	35.89	79.76	1.598	0.434
**T3**	45.00	6.330	38.67	85.93	1.962	0.353
**T4**	45.00	5.100	39.90	88.67	2.178	0.318

Values are means of three replicates; Co = Initial lindane concentration in polluted soil; Ct = Residual/final lindane concentration in soil; Loss = (Co−C_t_) in mg/kg; DE = Degradation efficiency; K_1_ = Degradation rate constant, t_1/2_ = half-life; T1-T4 = treatment levels 10% to 40% respectively.

The recorded initial and final concentration of lindane before and after the experiment was used to deduce the lindane degradation rate constant and its half-life in each treatment. Phytoremediation and mycoremediation were affirmed in controls 1 and 2 respectively. Degradation constant of 1.291 /day was recorded in control 1 and 1.371 was recorded in control 2, 1.313 and 1.598 were recorded in treatments 1 and 2 respectively while the T4 and T3 had the highest K_1_ of 2.178 and 1.962 /day (Table 3 above). The T4 and T3 had the half-life of 0.318 and 0.353 /day, 0.528 and 0.434 /day half-life was recorded in T1 and T2 respectively while 0.536 and 0.506 /day half-life was recorded in controls 1 and 2 respectively.

### Detection of phosphoesterases and catechol 1,2-dioxygenases genes in the fungal strains

**Fig 2:**
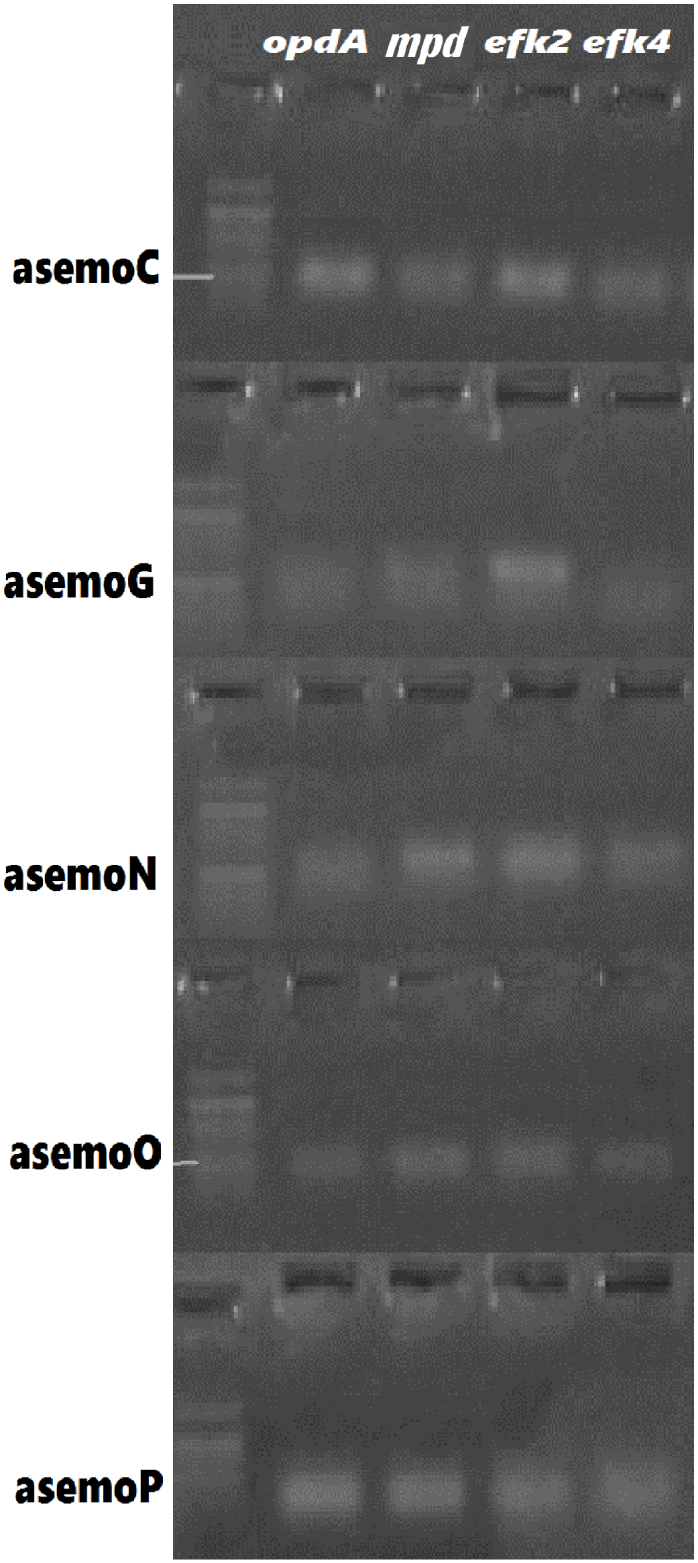
PCR Detection of genes encoding phosphoesterase and catechol 1,2-dioxygenase in in lindane utilizing fungal strains. *mpd* and *opd-A* = phosphoesterases; *afk2* and afk4 = catechol 1,2-dioxygenase; asemoC = *A*. *niger* KY693970; asemoG = *T*. *atroroseus* KY488464; asemoN = *T*. *purpurogenus* KY488468; asemoO = *Y*. *lipolytica* KY488469, and asemoP = *A*. *flavus* KY693973.

Detection of phosphoesterases (*mpd* and *opd-A*) genes and catechol 1,2-dioxygenase (*efk2* and *efk4*) genes in each of the fungal strains were detected on the gDNA of all the identified fungal strains through PCR gene amplification using specific primers for each gene (Table 1). The gel electrophoresis results of the PCR product showed that all the fungal strains *Aspergillus niger* asemoC KY693970, *Talaromyces atroroseus* asemoG KY488464, *Talaromyces purpurogenus* asemoN KY488468, *Yarrowia lipolytica* asemoO KY488469 and *Aspergillus flavus* asemoP KY693973 possess all the tested genes for production of phosphoesterase and catechol enzymes (Fig 2).

### Expression of phosphoesterase and catechol 1,2-dioxygenase genes in each fungal strains

**Fig 3:**
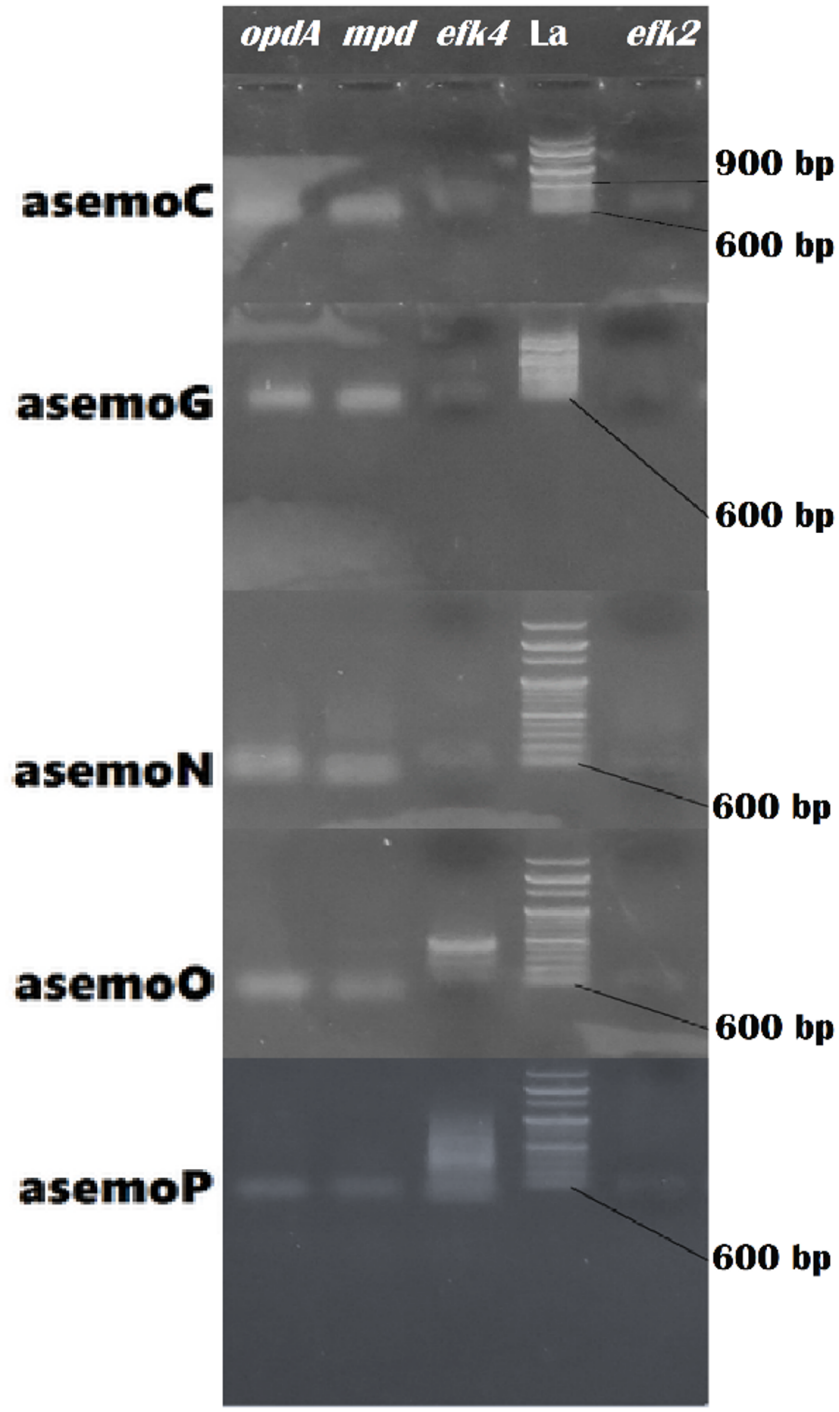
RT-PCR expression of genes encoding phosphoesterase and catechol 1,2-dioxygenase in lindane utilizing fungi. *mpd* and *opd-A* = phosphoesterases; *afk2* and *afk4* = catechol 1,2-dioxygenase; asemoC = *A*. *niger* KY693970; asemoG = *T*. *atroroseus* KY488464; asemoN = *T*. *purpurogenus* KY488468; asemoO = *Y*. *lipolytica* KY488469, and asemoP = *A*. *flavus* KY693973.

Gene expression of *opd*, *mpd*, *efk2 and efk3* were carried out on a synthesized cDNA from the mRNA of each fungal strain. In *Aspergillus niger* KY693970, there was overexpression of *opd* and *mpd*, while *efk4* and *efk2* where moderately expressed (Fig 3). There was also overexpression of *opd* and *mpd* in *Talaromyces atroroseus* KY488464 but *afk4* and *afk2* were under-expressed, this organism use more of phosphoesterase mechanism than catechol. *Talaromyces purpurogenus* KY488468 however showed an overexpression of *opd* and *mpd*, moderate expression of *efk4* and under-expression of *afk2* (Fig 3). Furthermore, *Yarrowia lipolytica* KY488469 showed overexpression of *opd* moderate expression of *efk4* and under-expression of *efk2* while *Aspergillus flavus* KY693973 shows moderate expression of *opd*, *mpd efk4* and *efk2* respectively.

## Discussion

This study affirms the rhizosphere degradation of lindane through fungi andplant (*M*. *maximus*) roots,genes coding associated fungal enzyme were also studied. Enzymes have been well reported to act as catalysts during biodegradation in many organisms [35], Generally, diverse bacteria and fungi has been reported to have developed adaptive mechanisms and ability to utilize/degrade many pesticides in soil, their ability to produce different catalytic enzymes not only as driver of their degradative mechanisms but also aid their tolerance ability [36, 37]. Also, enzymes may be produced by single microbial species in the soil or as a result of co-action of many soil microorganisms as controlled by several genes coding the secretions for protein enzyme which are of extensive and great variability with versatile catabolism [38]. Although large number of studies have been published regarding the isolation and characterization of bacterial and fungal strains for pesticide degradation or mineralization, but few reports have characterized their catabolic enzymes genes [39, 40].

In this study, synergistic rhizosphere degradation was shown through combine actions of fungi, SMC and plant root and this combined actions were compared with when they acted singly on the pesticide. The lindane dissipation over time was observed both in the controls (when the fungi and plant acted on lindane singly) and treatments (when the fungi and plant acted synergistically in the rhizosphere), this was however higher through combined actions of fungi and plant root. The use of SMC in this study with fungi also increased lindane degradation, this corroborated the reported of [23, 26, 27, 28, 41] that organic amendments can also enhance the soil remediation, especially degradation of lindane.

We also reported organophosphorus hydrolase enzymes encoded by phosphoesterase and catechol 1, 2- dioxygenase genes in the fungal strains, these enzymes has been reported widely in the degradation of wide range of organophosphorus insecticides such as organophosphorus, fenitrothion and methyl parathion. High and reasonable level of degradation rate constant (1.313–2.178 /day) and half-life (0.528–0.318) were recorded in treatments for lindane degradation in this experiment. Lindane half-life was previously estimated to take about 2.3 days in the atmosphere by Mackay et al. [42] and Brukaber and Hites [43] reported that lindane half-life takes 96 days in air. 3 to 30 days of lindane half-life was recorded for rivers, 30 to 300 days for lakes according to experimental hydrolysis half-life performed by Mackay et al. [41]. Shen et al. [44] earlier reported that about 12 to 30% of lindane may volatilizes into the atmosphere, Walker et al. [45] also estimated 580 pg per m^3^ of lindane in the atmosphere globally and this can be washed down by rainfall [46]. Adverse effects of lindane accumulation in soil on plant and microfloral has been reported many scientists [47–50], It can be absorbed into the food chain if not degraded in the soil or water due to its lipophilic characteristics [51].

The use and production of lindane was banned in 2005 under the Stockholm Convention on POPs [52], its use was as well banned in more than 50 countries and restricted in about 33 countries [53]. Unfortunately, the use of lindane still persist in many developing countries due to its famous and effect action as pesticide and illiteracy of many [54] for example, lindane residues in soil was detected in India according to the report of Agnihotri et al. [55], Titus et al. [56] and Nawab et al. [57]. High lindane residue above tolerant limit was also detected in food products and dairy milk according to the reports of [58, 59] respectively. Also, lindane concentration has been detected in ground-water, drinking water [60, 61], Prakash et al. [62] reported it in commercial brands of drinking water, Narain, [63] also reported it in soft drinks.

Generally, residual lindane concentrations in soil can be completely degraded using some microorganism, this has been reported in aerobic degradation pathway by bacterial strain *S*. *japonicum* (formerly *S*. *paucimobilis*) UT26 [64], this strain transformed lindane to 2,5-dichlorohydroquinone using the secreted enzyme actions controlled by genes LinA, B, and C and it was further metabolize it to succinyl-CoA and acetyl-CoA through catalytic control of LinD, LinE, LinF, Lin GH and Lin J through the citrate/tricarboxylic acid cycle [64]. Another lindane degradation pathway by *S*. *japonicum* was presented by Quintero et al. [65] and Endo et al. [66] as the organism dechlorinated lindane to form pentachlorocyclohexane, and finally to mono-chlorobenzene. We recorded tetrachlorocyclohexene and tetrachlorocyclohexenol as metabolites of lindane degradation in the soil experiment, this corroborates the reports of [67, 68] during the degradation of lindane by *Phanerochaete* spp and the report of Manickam et al. [69] during the degradation of lindane by *Xanthomonas* sp. The degradation of pesticides in soil is a function of their bioavailability to microorganisms, microbial population, their activities and enzymatic systems [70]. SMC commonly applied as soil amendments helps these factors a lot and improves soil productivity [71]. Addition of organic amendments often changes the pathways of pesticide movement and degradation in soils, depending on the reactivity of the organic amendments and their effect on microbial activity [72, 73]. Earlier studies have also confirmed that organic amendments enhanced the degradation of lindane in soil [74–76].

**Fig 4:**
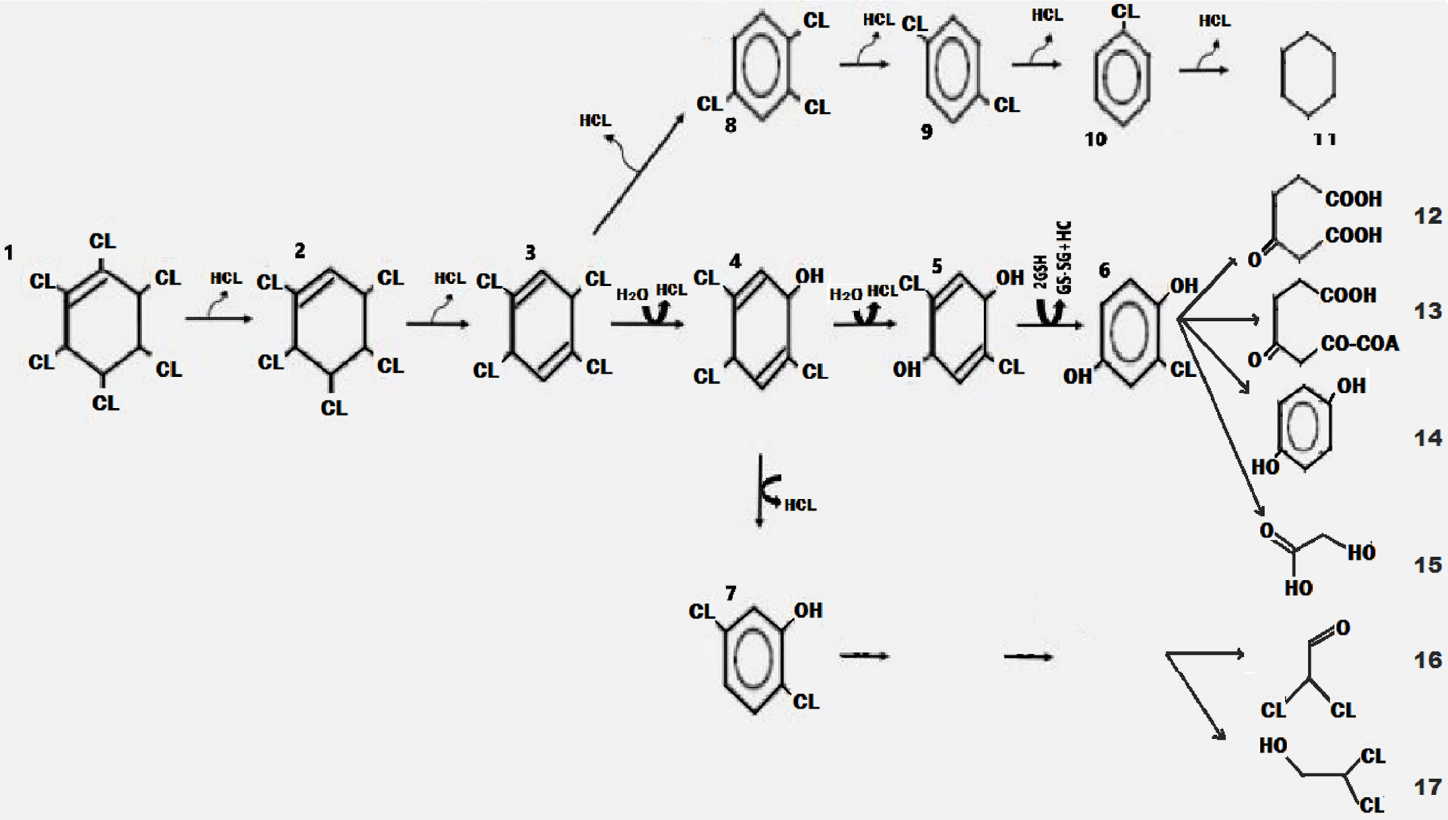
Proposed degradation of γ-Hexachlorocyclohexane (lindane) through synergistic rhizosphere degradation by the rhizospheric fungal strains and *M*. *maximus*. Compounds: 1. (γ-Hexachlorocyclohexane) 2. Pentachlorocyclohexene (γ-PCCH) 3. 1,3,4,6-Tetrachloro-1,4-cyclohexadiene (1,4-TCDN) 4. 2,4,5-Trichloro- 2,5-cyclohexadiene-1-ol (2,4,5-DNOL) 5. 2,5-Dichloro-2,5-cyclohexadiene-1,4-diol (2,5-DDOL) or 2,5-Dichlorohydroquinone (2,5-DCHQ), 6. Chlorohydroquinone (CHQ) 7. 2,5-Dichlorophenol (2,5- DCP) 8. 1,2,4-Trichlorobenzene (1,2,4-TCB) 9. 2,5-Dichlorobenzene (2,5-DCB) 10. Chlorobenzene 11. benzene 12. β-Ketoadipate (3-oxoadipate) 13. 3-Oxoadipyl-CoA 14. γ-Hydroxymuconic semialdehyde 15. Glycolic acid 16. Dichloroacethaldehide 17. Dichloroethanol.

In this study also, the analyzed experimented soil through the use of GC/MS (S3 Table) gave an insight into lindane degradation pathway due to initial and final concentrations of detected lindane metabolites in the soil. An aerobic degradation for lindane was then suggested as given in Fig 4 below.

Synergistic rhizosphere degradation of lindane using fungi and *M*. *maximus* root, follows the following three proposed pathways:

1. The dechlorination of γ-HCH (lindane) into (γ-Hexachlorocyclohexane), till (2,5-DCB), Chlorobenzene and benzene (Fig 4).
2. The dechlorination of γ-HCH (lindane) into (γ-Hexachlorocyclohexane), Pentachlorocyclohexene (γ-PCCH) and 1,3,4,6-Tetrachloro-1,4-cyclohexadiene (1,4-TCDN) followed by its hydration into 2,4,5-Trichloro- 2,5-cyclohexadiene-1-ol (2,4,5-DNOL) and 2,5-Dichloro-2,5-cyclohexadiene-1,4-diol (2,5-DDOL) or 2,5-Dichlorohydroquinone (2,5-DCHQ) till it forms Glycolic acid (Fig 4). The end product of this pathway have been earlier reported in some aerobic degradation study to form Chlorohydroquinone (CHQ) which is then degraded into β-Ketoadipate (3-oxoadipate), 3-Oxoadipyl-CoA and γ-Hydroxymuconic semialdehyde in many bacteria [64, 69, 77].
3. The dechlorination of γ-HCH (lindane) into (γ-Hexachlorocyclohexane), Pentachlorocyclohexene (γ-PCCH) and 1,3,4,6-Tetrachloro-1,4-cyclohexadiene (1,4-TCDN) followed by subsequent enzymatic degradation into 2,4,5-Trichloro- 2,5-cyclohexadiene-1-ol (2,4,5-DNOL), 2,5-Dichlorophenol (2,5- DCP), Dichloroacethaldehide and Dichloroethanol (Fig 4).

These proposed pathways resemble that of Nagata et al. [78] which was proposed for *Sphingobium japonicum* degradation of lindane, has as reportedly controlled by LinD, LinE, LinF, LinGH and LinJ which enable the organism to metabolized lindane to succinyl-CoA and acetylCoA, in the citrate/tricarboxylic acid cycle. These pathways also resemble reported dechrorination of lindane by this organism as reported by Endo et al. [66]. The pathways presented here all started with the dechlorination of α- and γ-HCH is to form pentachlorocyclohexane, which the degraded to form 1,2-dichlorobenzene (DCB) and 1,3-dichlorobenzene isomers, chlorobenzene and benzene in pathway (1) above, this was similarly reported by Quintero et al. [64]. Degradation of lindane intermediate as mentioned in pathways (ii) and (iii) above have been earlier reported by different scientists [79, 80, 81] and formation of ethanone 1-(3-chloro-4-methoxyphenyl)- and 1-benzenecarbonyl chloride, 2,4-dichloro-3- methoxy organochlorines from the lindane degradation by fungus *Sphingobium indicum* B90A were earlier reported by [64, 69, 78]. Reports of Nagata et al. [65] and Endo et al. [66] showed that lindane can be mineralized into 1, 2, 4-TCB, 2,5-DCP and 2,5-DCHQ by enzymatic actions of dehydrochlorinase enzyme which is controlled by *LinA* gene [82], halidohydrolase enzyme through *LinB* gene [79] and dehydrogenase enzyme controlled by *LinC* gene [83] these steps by them were referred to as ‘upstream pathway’ and in these pathways, formation of 2,5-DCHQ was reported to be controlled by reductive enzyme dechlorinase which was reported to be controlled by a gene called LinD [84], ring-cleavage dioxygenase enzyme controlled by LinE gene [84], and a maleylacetate reductase enzyme which is controlled by LinF gene [85].

Nagata et al. [82, 83] and Miyauchi et al. [84] had also successfully expressed these linA, linB, and linC genes which are believed to take critical roles in upstream pathway in lindane degradation in this bacterium, some other putative regulatory gene were also reported [86, 87]. In this study however, all the five selected fungal strains possess the tested phosphoesterases and catechol 1,2-dioxygenases genes, they all however showed good expression of phosphoesterase gene over the catechol gene which suggests that the fungi would produce more of phosphoesterases. These also suggest that fungal degradation of lindane by fungi could be as a result of different cassettes of enzymes.

## Conclusion

The degradation of lindane through synergistic rhizosphere degradation was reported in this work for the first time. Combined treatments of lindane polluted soil with the selected fungi, SMC and *M*. *maximus* root speed up lindane degradation kinetic within three months and a possible pathway for lindane degradation was suggested based on the residual lindane metabolites detected in the soil. The comparison between single lindane biodegradation by plant or fungi alone was compared with synergistic lindane degradation and we observed that lindane degradation in polluted soil was more effective when the actions of plant’s root and fungi were combined. In selected fungal strains, the genes encoding the enzymes phosphoesterases and catechol 1,2-dioxygenase were detected and expressed. These enzymes may be associated in the degradation of lindane and expressed in the selected fungal strains *A*. *niger* asemoC KY693970, *T*. *atroroseus* asemoG KY488464, *T*. *purpurogenus* asemoN KY488468, *Y*. *lipolytica* asemoO KY488469 and *A*. *flavus* asemoP KY693973. There is a need of large scale, more in-depth, evaluation of bioremediation protocols especially using these fungal strains and this hold a potential headway for lindane polluted soil remediation.

## Supporting information

S1 Table Isolated rhizospheric fungal strains from lindane polluted soil with their percentage incidences. (DOCX)

S2 Table Molecular identification of rhizospheric fungal strain with accessions. (DOCX)

S3 Table Effect of synergistic rhizosphere degradation of lindane and its metabolites in polluted soil. (DOCX)

S1 Fig Gel electrophoresis analysis of PCR product for the ITS gene fragment. ITS gene amplification in asemoC (C), asemoG (G), asemoN (N), O asemoO (O) and in asemoP (P).
(TIF)
