# Supplementary material for: Synergistic rhizosphere degradation of γ-hexachlorocyclohexane (lindane) through the combinatorial plant-fungal action
Source: PLoS One. 2017 Aug 31;12(8):e0183373. doi: 10.1371/journal.pone.0183373 (PMC5578508; doi:10.1371/journal.pone.0183373)
Supplement: S1 Table — (DOCX) [file pone.0183373.s001.docx]

**Table S1:** Isolated rhizospheric fungal strains from lindane polluted soil with their percentage incidences.

| **Fungal Species Code** | **SOIL 4 (N=50)** | **% Incidence** |
| --- | --- | --- |
| asemoA | 03 | 06 |
| asemoB | 04 | 08 |
| asemoC | 41 | 82 |
| asemoD | 02 | 42 |
| asemoG | 28 | 56 |
| asemoH | 12 | 24 |
| asemoI | 23 | 46 |
| asemoJ | 02 | 04 |
| asemoK | 12 | 24 |
| asemoL | 12 | 24 |
| asemoM | 07 | 14 |
| asemoN | 28 | 56 |
| asemoO | 34 | 68 |
| asemoP | 34 | 68 |
| TNIT | 271 |  |

N = Total number of fungi isolated; TNIT = Total number of Incident Time, Only isolate with more than 50% percentage incidence were selected for further studies.
