# Supplementary material for: Synergistic rhizosphere degradation of γ-hexachlorocyclohexane (lindane) through the combinatorial plant-fungal action
Source: PLoS One. 2017 Aug 31;12(8):e0183373. doi: 10.1371/journal.pone.0183373 (PMC5578508; doi:10.1371/journal.pone.0183373)
Supplement: S2 Table — (DOCX) [file pone.0183373.s002.docx]

**Table S2:** Molecular identification of rhizospheric fungal strain with accessions.

| **Isolate Code** | **NCBI Submission ID** | **Accession no** | **Name** | **BLAST**  **Percentage**  **Similarity** |
| --- | --- | --- | --- | --- |
| AsemoC | 1997057 | KY693970 | *Aspergillus niger* | 97% |
| AsemoG | 1984212 | KY488464 | *Talaromyces atroroseus* | 99% |
| AsemoN | 1984212 | KY488468 | *Talaromyces purpurogenus* | 98% |
| AsemoO | 1984212 | KY488469 | *Yarrowia lipolytica* | 99% |
| AsemoP | 1997057 | KY693973 | *Aspergillus flavus* | 98% |
