# Supplementary material for: Synergistic rhizosphere degradation of γ-hexachlorocyclohexane (lindane) through the combinatorial plant-fungal action
Source: PLoS One. 2017 Aug 31;12(8):e0183373. doi: 10.1371/journal.pone.0183373 (PMC5578508; doi:10.1371/journal.pone.0183373)
Supplement: S3 Table — (DOCX) [file pone.0183373.s003.docx]

**Table S3:** Effect of synergistic fungi and root treatments on Lindane (mg/kg) content in soil 4 after 3 months

| **Pesticide** | **mol. Weight** | **Initial (mg/kg)** | **Control 0%** | | | **T1 (10%)** | | | **T2 (20%)** | | | **T3 (30%)** | | | **T4 (40%)** | | |
| --- | --- | --- | --- | --- | --- | --- | --- | --- | --- | --- | --- | --- | --- | --- | --- | --- | --- |
|  | **(g/mol)** | **C_0_** | **C_t_** | **Loss** | **% Loss** | **C_t_** | **Loss** | **% Loss** | **C_t_** | **Loss** | **% Loss** | **C_t_** | **Loss** | **% Loss** | **C_t_** | **Loss** | **% Loss** |
| Lindane | 220.0 | 45.00 | 12.34 | 32.66 | 72.58 | 12.11 | 32.89 | 73.09 | 9.11 | 35.89 | 79.76 | 6.330 | 38.67 | 85.93 | 5.100 | 39.90 | 88.67 |
| Other detected residual compounds |  |  |  |  |  |  |  |  |  |  |  |  |  |  |  |  |  |
| 2,5-Dichlorobenzene (2,5-DCB) | 192.0 | 13.24 | 14.23 |  |  | 16.23 |  |  | 18.34 |  |  | 23.32 |  |  | 34.32 |  |  |
| 2,4-Dichlorophenol | 162.9 | 8.450 | 10.34 |  |  | 12.32 |  |  | 11.23 |  |  | 18.23 |  |  | 18.17 |  |  |
| Pentachlorocyclohexene | 256.4 | 11.26 | 13.32 |  |  | 18.94 |  |  | 19.32 |  |  | 17.23 |  |  | 17.11 |  |  |
| Chlorobenzene | 112.6 | 9.340 | 23.21 |  |  | 19.34 |  |  | 23.24 |  |  | 30.43 |  |  | 45.32 |  |  |
| Pentachlorocyclohexanone | 270.7 | 11.23 | 34.4 |  |  | 36.98 |  |  | 67.56 |  |  | 68.45 |  |  | 65.34 |  |  |

*Values are means of three replicates;* *Co = Concentration of the initial lindane (mg/kg); Ct = Concentration of the final lindane (mg/kg) and Loss = (C_o_ - C_t_) in mg/kg; Biodegradation efficiency (BE) is calculated as percentage loss to determine the efficiency of each treatment; T1-T4 = soil treated with 10-40 % SMC-Fungal treatments*.
