# Supplementary figures and images for: Synergistic rhizosphere degradation of γ-hexachlorocyclohexane (lindane) through the combinatorial plant-fungal action
Source: PLoS One. 2017 Aug 31;12(8):e0183373. doi: 10.1371/journal.pone.0183373 (PMC5578508; doi:10.1371/journal.pone.0183373)

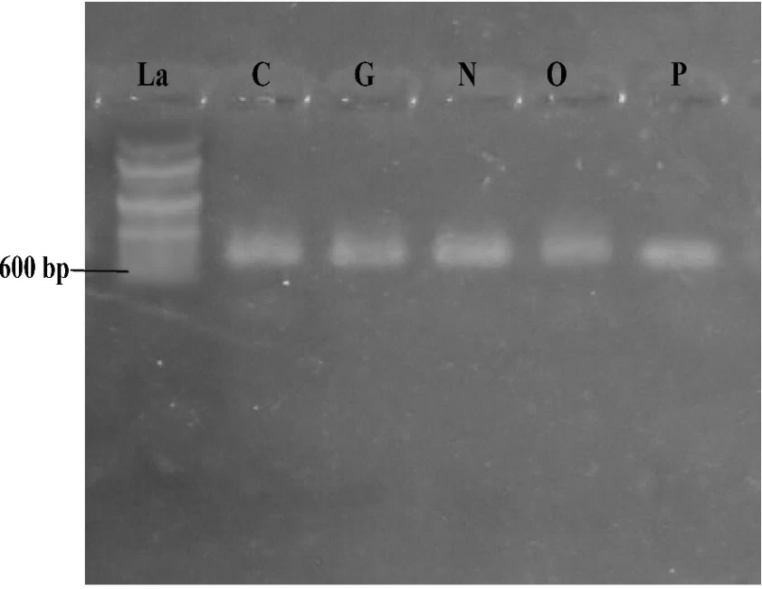

Supplement: S1 Fig — ITS gene amplification in asemoC (C), asemoG (G), asemoN (N), O asemoO (O) and in asemoP (P). (TIF) [file pone.0183373.s004.tif]
